# SUN11602 Accelerates Early Peripheral Nerve Regeneration in an Experimental Median Nerve Transection Model

**DOI:** 10.1111/jcmm.71375

**Published:** 2026-09-26

**Authors:** Giovanna Casili, Igor Papalia, Alessio Ardizzone, Rossella Basilotta, Alfio Luca Costa, Paolo Titolo, Emanuela Esposito, Michele Rosario Colonna, Mariarosaria Galeano

**Affiliations:** ^1^ Department of Chemical, Biological, Pharmaceutical and Environmental Sciences University of Messina Messina Italy; ^2^ Department of Biological and Morphological Sciences and Imaging University of Messina Messina Italy; ^3^ Departmental Faculty of Medicine UniCamillus‐Saint Camillus International University of Health Sciences Rome Italy; ^4^ Clinic of Plastic Surgery, Department of Neurosciences University of Padua Padua Italy; ^5^ Reconstructive Microsurgery Unit, Department of Orthopedics & Traumatology, AOU Città della Salute e della Scienza di Torino Turin Italy; ^6^ Department of Human Pathology of Adult and Childhood “G. Barresi” University of Messina Messina Italy

## Abstract

Peripheral nerve injury (PNI) is one of the common life‐altering disabilities resulting in various degrees of impairment in movement and sensation alongside apraxia and long‐lasting pain. The gold standard for restoring the transected nerves is bridging the distal and proximal ends of disrupted autologous nerve using neurosurgical sutures. This approach is helpful, however nerve bridging is valid in pay of consuming healthy nerves and scarring phenomena in damaged site besides difficult donor nerve accessibility and extra surgery is required. Many studies have demonstrated that bFGF plays a pivotal role in the regeneration of PNI and severe spinal cord injury, exerting neuroprotective activity by binding to fibroblast growth factor receptors (FGFRs). Particularly, bFGF exhibits a high affinity for the receptor FGFR1, serving as its specific ligand. The novel synthetic compound SUN11602 exhibited neuroprotective activities like bFGF. Therefore, this study aimed to evaluate the effect of SUN11602 treatment in a rat experimental model of peripheral median nerve transection (MNT) and end‐to‐end suture. MNT was performed on rats, starting from 24 h following the surgical procedure, to orally treat, daily for 7 days, with SUN11602 at the dose of 2.5 mg/kg. The results showed that SUN11602 administration significantly reduced the alteration of PNI, promoting median nerve regeneration, suppressed mast cell accumulation, and resident macrophages. Also, SUN11602 reduced pro‐inflammatory response of M1 macrophages, stimulated Schwann cell proliferation, and slowed down median nerve demyelination and fibrosis. Therefore, SUN11602 could be considered a valuable pharmacological strategy for MNT.

## 1 Introduction

Peripheral nerve injury (PNI) can be caused by various factors such as inflammation, trauma, entrapment, mechanical damage, tumour radiotherapy and chemotherapy [1]. The peripheral nervous system (PNS) has a limited endogenous regenerative capacity following injury, leading to incomplete restoration of motor and sensory function that affects overall quality of life [2]. Following PNI, the distal nerve fibres undergo Wallerian degeneration, characterized by degradation of axoplasm and axolemma, accompanied by production of myelin debris that must be removed prior to regeneration [3]. Degeneration and subsequent regeneration of the peripheral nerve require extensive coordination of multiple cell types, beginning with the breakdown of axons and myelin sheaths [4]. Schwann cells (SCs) in the distal nerve undergo a rapid de‐differentiation into non‐myelinating repair stem cells that secrete pro‐regenerative factors, clear myelin directly through autophagy and indirectly by activating the innate immune response, recruit macrophages and form regeneration tracks onto which newly growing axons can be guided from the proximal stump to their original target tissues [5, 6, 7]. Macrophages also play a variety of roles in the processes of neuronal degeneration and regeneration, such as the cell debris removal and axon elongation [8]. Treatment of peripheral nerve injuries is one of the most important challenges in clinical practice [9]. Nowadays, microsurgical techniques have greatly improved peripheral nerve repair procedures [10]. However, the nerve regeneration rate is relatively slow compared to other tissues, and it may take up to 10 months for axons to reach the target tissues. Therefore, the treatment should be used for a long time. Neurotrophic substances and neurotrophins have an important role in nerve regeneration [11], but only a few of them can be used in clinical practice due to their side effects and high costs [12]. In experimental studies on animals, the effects of various pharmacological agents on nerve healing continue to be examined. Despite many experimental studies, a treatment method that allows the peripheral nerve to function normally has not been found yet.

Basic fibroblast growth factor (bFGF; FGF‐2), a key member of the FGF family, is one of the powerful neurotrophic factors secreted by SCs and different neuronal populations [13, 14]. Several experiments have demonstrated that bFGF can stimulate the proliferation and differentiation of brain neurons [15], SCs, fibroblasts, and plays a pivotal role in the regeneration of PNI and severe spinal cord injury [16, 17, 18, 19, 20]. bFGF exerts neuroprotective activity by binding to fibroblast growth factor receptors (FGFRs), a group of transmembrane tyrosine kinase receptors that transmit FGF signals into cells [21]. In particular, bFGF exhibits a high affinity for the receptor FGFR1, serving as its specific ligand [22]. A clinical trial using native bFGF in acute stroke patients failed to demonstrate therapeutic efficacy and full safety. In particular, the use of bFGF caused fever, decreased blood pressure, increased leukocyte counts and carcinogenesis [23, 24]. Therefore, it was necessary to synthesize bFGF functional analogues in order to create safer drugs that mimic the beneficial effects of bFGF. SUN11602 is a novel aniline derivative that exhibits bFGF‐like comparable neuroprotective effects without promoting proliferative activity during treatment [25]. SUN11602 interacts with the FGFR1 receptor similarly to bFGF, consequently stimulating neuronal growth and survival in pathological conditions [25, 26]. Moreover, the FGFR1 activation induced by SUN11602 increases the expression of Calbindin‐D28k, a Ca^2+^ binding protein expressed in several areas of the brain and in the maintenance of Ca^2+^ intracellular homeostasis. The pharmacokinetic properties of SUN11602 appear to hold promise in terms of bioavailability (> 65%) after oral administration in rodents and dogs [26, 27].

Several neurotrophic factors, including NGF, BDNF, GDNF and NT‐3, have been extensively investigated for their ability to promote neuronal survival and axonal regeneration following PNI [28, 29, 30, 31]. In addition, other growth factors such as VEGF and IGF‐1 have demonstrated supportive roles in nerve repair through angiogenic and neuroprotective mechanisms [32, 33]. However, their clinical application is often limited by poor pharmacokinetic properties, high costs and potential adverse effects [34]. In this context, small molecules mimicking neurotrophic activity represent a promising alternative. SUN11602 was selected for this study due to its ability to reproduce the neuroprotective effects of bFGF while exhibiting improved safety and bioavailability profiles as previously detailed. Thus, compared to other neurotrophic strategies, this compound offers the advantage of oral administration and reduced side events, making it a particularly attractive candidate for translational applications. Therefore, in this study we evaluated the possible neuroprotective role of SUN11602 in an experimental model of median nerve transection (MNT) and end‐to‐end suture.

## 2 Materials and Methods

### 2.1 Materials

SUN11602 was purchased from Tocris Bioscience company (Bristol, UK). SUN11602 was dissolved in 10% Dimethyl Sulfoxide (DMSO) and diluted in a saline solution according to manufacturer instructions. Unless specified differently, all compounds used in the experiment were obtained from Sigma‐Aldrich Company Ltd. (Milan, Italy). All the chemicals were of the highest commercial grade available. All stock solutions were prepared in non‐pyrogenic saline (0.9% NaCl; Baxter, Italy, UK).

### 2.2 Animals

Six‐week‐old female Sprague–Dawley rats (200–250 g) were purchased from Envigo (Italy). The rats were acclimated to standard laboratory conditions with a light/dark cycle of 12 h and a temperature of 22°C ± 1°C. Rats were fed with a standard diet and water and monitored for 1 week in quarantine prior to the start of the experiment. This study was performed following Italian regulations on the use of animals (D.M.116192) and Directive legislation (EU) (2010/63/EU) amended by Regulation (EU) 2019/1010 as well as ARRIVE guidelines. In addition, the study was approved by the Ministry of Health with the authorization number 856/2023‐PR.

### 2.3 Surgical Model of MNT and End‐to‐End Suture

All surgical procedures were carried out in the Laboratory of Pharmacology, University of Messina.

Sixteen adult Wistar female rats weighing 250–300 g were used in this study. In the animals, under general anaesthesia by ketamine (40 mg/250 g) and cloropromazine (3.75 mg/250 g) and clean conditions, the median nerve of the left upper limb was approached from the axillary region and carefully exposed at the middle third of the brachium and transected, under an operative microscope. The severed median nerve was then repaired by end‐to‐end suture by means of 2–3 epineurial stitches of 9–0 monofilament nylon.

Starting from 24 h following the surgical procedure, six rats, randomly selected, were treated daily with SUN11602 at the dose of 2.5 mg/kg orally via gastric gavage; the administrations were performed for 7 days. The dose and the route of administration of SUN11602 used in this study were based on earlier in vivo investigations carried out in our laboratory [35, 36]. 24 h after the end of treatment with SUN11602 2.5 mg/kg, rats were euthanized via anaesthesia overdose followed by cervical dislocation, nerve tissues were surgically removed and collected for histological examinations.

**FIGURE 1:**
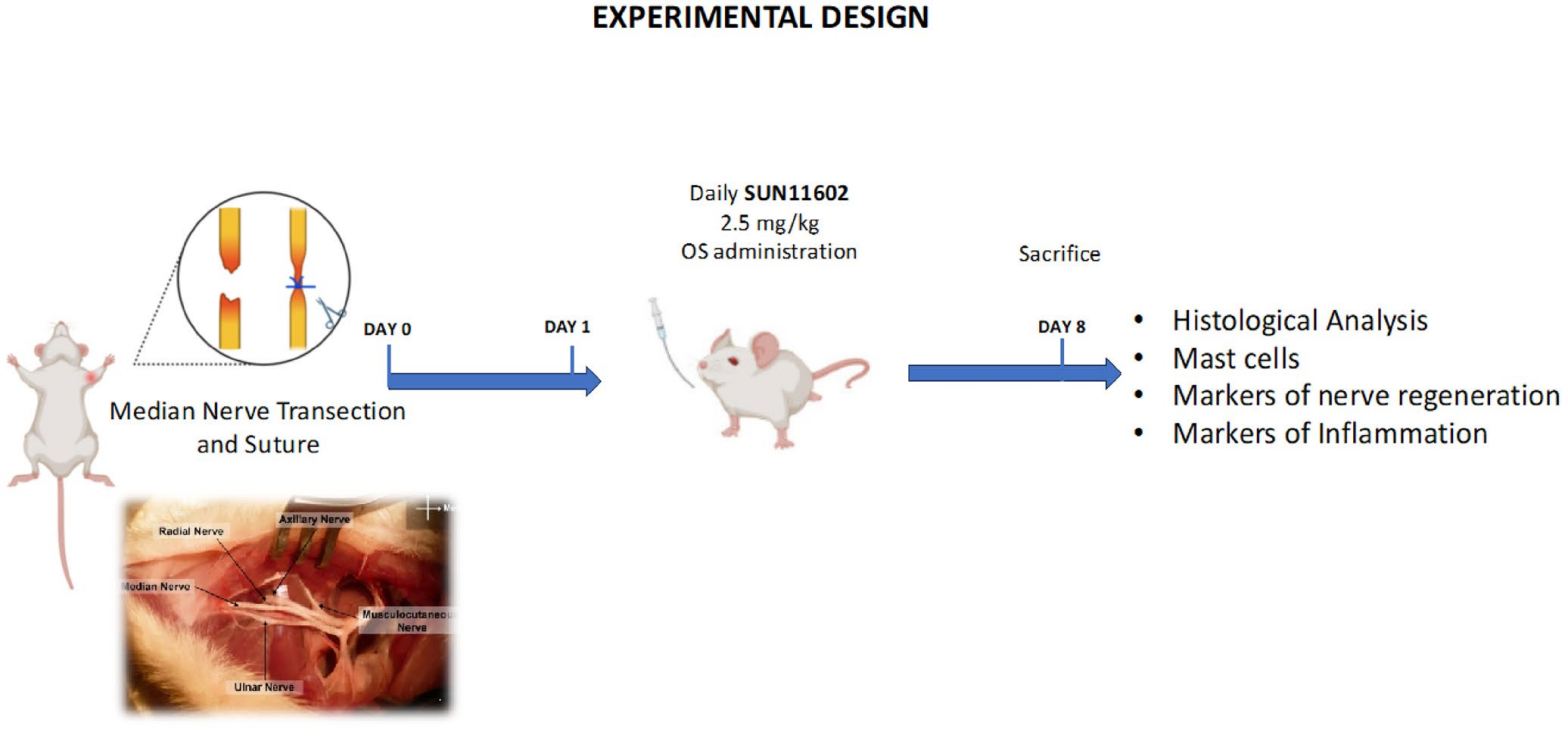
Schematic summary of the experimental protocol adopted in this study.

A graphical representation of the experimental design is provided in Figure 1.

### 2.4 Experimental Groups

Prior to starting the experiment, the animals were allocated randomly into the three following groups:

Group 1: Sham + vehicle: Animals underwent surgical incision without executing nerve; 24 h following surgical procedure, vehicle (saline) was administered once daily via oral gavage for 7 consecutive days (*N* = 6).

Group 2: MNT + vehicle: Animals underwent surgical incision with median nerve section and end‐to‐end neurorrhaphy; 24 h following the surgical procedure, vehicle (saline) was administered once daily via oral gavage for 7 consecutive days (*N* = 6).

Group 3: MNT + SUN11602 2.5 mg/kg: Animals underwent surgical incision with median nerve section and end‐to‐end neurorrhaphy, 24 h following surgical procedure SUN11602 2.5 mg/kg was administered once daily via oral gavage for 7 consecutive days (*N* = 6).

### 2.5 Histological Evaluation

The procedure employed for haematoxylin/eosin (H&E) staining was based on previous protocols [37]. After sacrifice, the nerve tissue samples were preserved in a 10% weight/volume (w/v) PBS‐buffered formaldehyde solution for 24 h at 25°C. The tissues were then gradually dehydrated with ethanol and fixed in paraffin. Thereafter, the samples were cut by microtome into 7 μm‐thick sections and fixed on poly‐L‐lysine coated glass slides. Then, H&E staining was performed to evaluate tissue morphology. A Nikon Eclipse Ci‐L microscope (NIKON CORPORATION, Tokyo, Japan) was used to acquire histological images, which were then shown at 2× (500 μm scale bar) and 20× magnifications (50 μm scale bar).

### 2.6 Toluidine Blue Staining

One of the most used methods for finding mast cells is toluidine blue staining, which was performed as previously described [38] and briefly reported below. The tissue slices were dehydrated, then embedded in paraffin (Bio‐Optica, Milan, Italy), and cut with a microtome into 7 μm sections. The slices were then gently blotted after being deparaffinized in xylene, dried in increasing ethanol concentrations until they were almost dry, and stained with toluidine blue for 3 min and mounted with Eukitt (Bio‐Optica, Milan, Italy) following dehydration. A Nikon Eclipse Ci‐L microscope was used to count and observe the mast cells on each slice; images are shown at a 40× magnification (20 μm scale bar).

### 2.7 Immunohistochemical Localization of IBA‐1

Immunohistochemical staining was performed in accordance with our previous study [39]. In brief, nerve sections were deparaffinized and hydrated in graded ethanol concentrations until water. Antigen retriever was performed in citrate buffer, and peroxidase activity was blocked using 3% H_2_O_2_. Tissues were then blocked with normal horse serum for 10 min, followed by incubation with primary anti‐IBA‐1 (1:100; sc‐32725; Santa Cruz Biotechnology) at room temperature for 3 h. Next, using the VECTASTAIN Universal Quick Kit, Peroxidase, R.T.U. (PK‐7800; Vector Laboratories, Burlingame, CA, USA), the sections were incubated with a secondary antibody after being cleaned with PBS. Brown DAB reaction was used to observe the immunoreactivity, and Nuclear Fast Red was used as a counterstain. A Nikon Eclipse Ci‐L microscope was used for blind examination and analysis of all stained sections. The findings of the immunohistochemistry were shown at 20× and 40× magnification, with a scale bar of 50 and 20 μm, respectively.

### 2.8 Immunofluorescence Analysis of S100 and CD68

Immunofluorescence staining of Nestin, S100, αSMA and β3 tubulin was conducted as previously indicated [35]. Thereafter deparaffinization, the following primary antibodies were incubated overnight on the nerve tissues: S100 (1:100; sc‐393919 Santa Cruz Biotechnology, Santa Cruz, CA, USA) and CD68 (sc‐20060, Santa Cruz). The day after, secondary fluorescent antibodies Alexa Flour 488 goat anti‐mouse (A11001; Molecular Probes, Eugene, OR, USA) or anti‐rabbit Alexa Fluor 594 (A11008; Molecular Probes, Eugene, OR, USA) were incubated for 3 h based on the primary antibody applied before. Afterward, slices were stained with 4′,6′‐diamidino‐2‐phenylindole (DAPI; Hoechst, Frankfurt, Germany) for nuclei revelation. The histology investigations were conducted in a blinded fashion, and the images were captured using a fluorescent microscope (Nikon Eclipse Ci‐L microscope, NIKON CORPORATION, Tokyo, Japan). Images are displayed at a magnification of 20× (50 μm scale bar).

### 2.9 MCOLL Staining

MCOLL staining was performed as previously described [40]. In short, the paraffin sections were hydrated in 100% and 95% ethanol and incubated with LFB (04‐210923 Bio‐Optica) at 56°C 24 h (overnight). The sections were rinsed in 95% ethanol and then in distilled water and differentiated into 0.05% lithium carbonate until it turns grey and white matter can be distinguished. The slides were rinsed with 70% ethanol and then in distilled water 5 min.

After the sections were coloured with 0.2% Sirius red (04‐200812 Bio‐Optica) in a saturated solution of picric acid for 90 min at room temperature. Finally, the counterstaining with Mayer's haematoxylin was carried out for 3 min. With MCOLL histochemical method it was possible to accurately identify the Nodes of Ranvier and the organization of the collagen fibres in the connective tissue surrounding the nerve fibres and blood vessels.

### 2.10 Statistical Analysis

The values in this study are given as the mean ± standard deviation (SD) of *N* observations, where *N* is the number of animals examined. A One‐way ANOVA was utilized to examine all the data followed by a Bonferroni post hoc test for multiple comparisons through the use of GraphPad Prism version 9.1.0 software. In all examinations only a *p*‐value of < 0.05 was deemed significant.

## 3 Results

### 3.1 SUN11602 Treatment Promoted Median Nerve Regeneration

**FIGURE 2:**
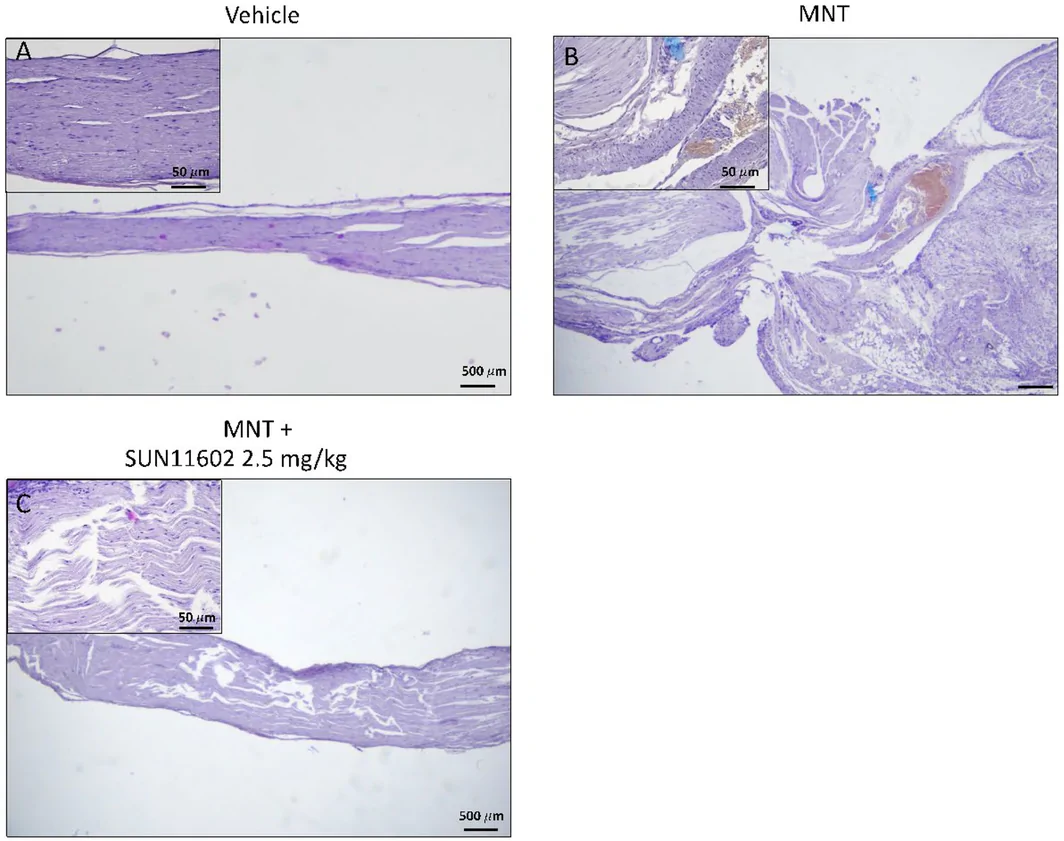
Haematoxylin and eosin staining on sagittal sections of median nerve: vehicle group (A), MNT group (B), SUN11602‐treated group (C). Data are representative of at least three independent experiments. The sections were observed and photographed at 2× and 20× magnification.

To evaluate the early histopathological effects of SUN11602 following PNI, haematoxylin and eosin staining was performed on sections of the rat median nerve at 7 days post‐suture. In the MNT group, we observed evident structural disorganization, oedema and signs consistent with early myelin and axonal degeneration, as expected during the acute phase of Wallerian degeneration (Figure 2B), compared to the vehicle group (Figure 2A). In contrast, nerves from the SUN11602‐treated group showed a comparatively more preserved architecture, with reduced vacuolization and less apparent tissue disarray (Figure 2C).

### 3.2 SUN11602 Treatment Suppressed Mast Cell Accumulation in Median Nerve Transection

**FIGURE 3:**
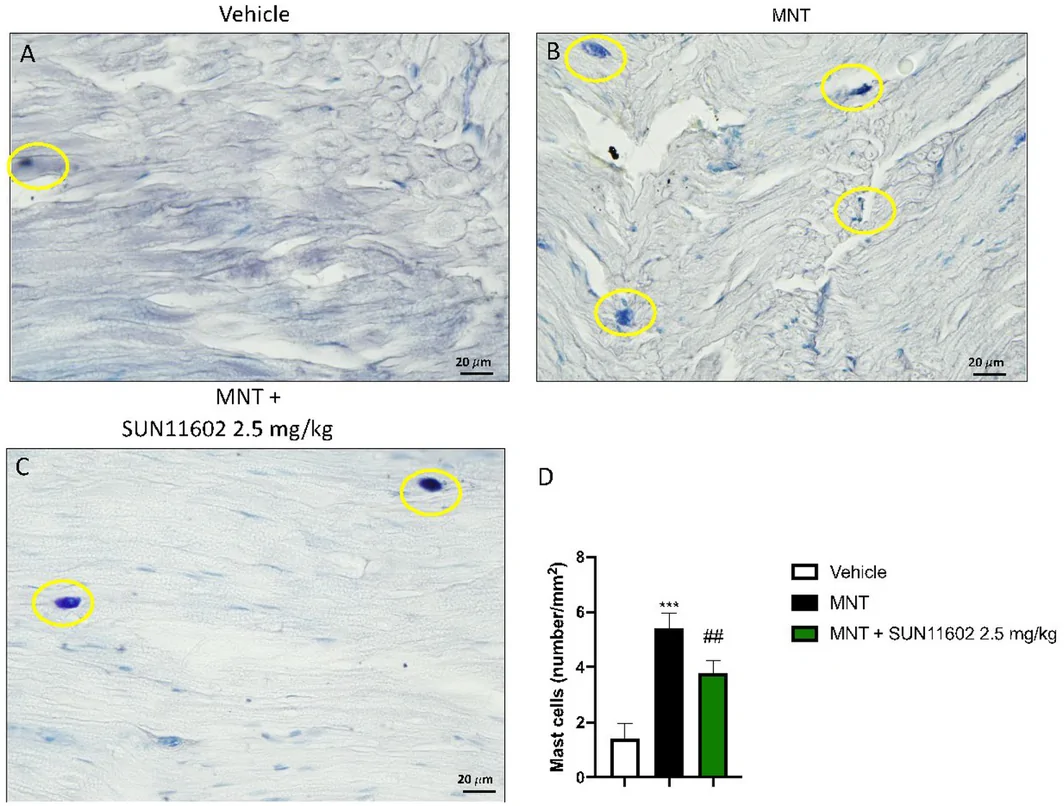
Toluidine blue staining on sagittal sections of median nerve: vehicle group (A), MNT group (B) SUN11602‐treated group (C), score (D). The data are representative of at least three independent experiments. The sections were observed and photographed at 40× magnification. ***p < 0.001 vs. vehicle; ##*p* < 0.01 vs. MNT.

Mast cells play an important role in nerve injury, contributing to chronic inflammation and pain and influencing the behaviour of other immune cells, SCs and the neurons themselves [41, 42]. Toluidine blue‐stained sections were used to determine the numbers of axons and mast cells. The MNT group showed increased numbers of mast cells in the connective tissue and axon fibres with irregular morphology and degenerated myelin sheath compared to the vehicle group (Figure 3A,B). These conditions were recovered by SUN11602 treatment at the dose of 2.5 mg/kg, as shown by the increase in axon fibre density due to the suppression of mast cell accumulation (Figure 3B,C, see score D).

### 3.3 SUN11602 Treatment Reduced Marker for Activated Macrophages in Median Nerve Transection

**FIGURE 4:**
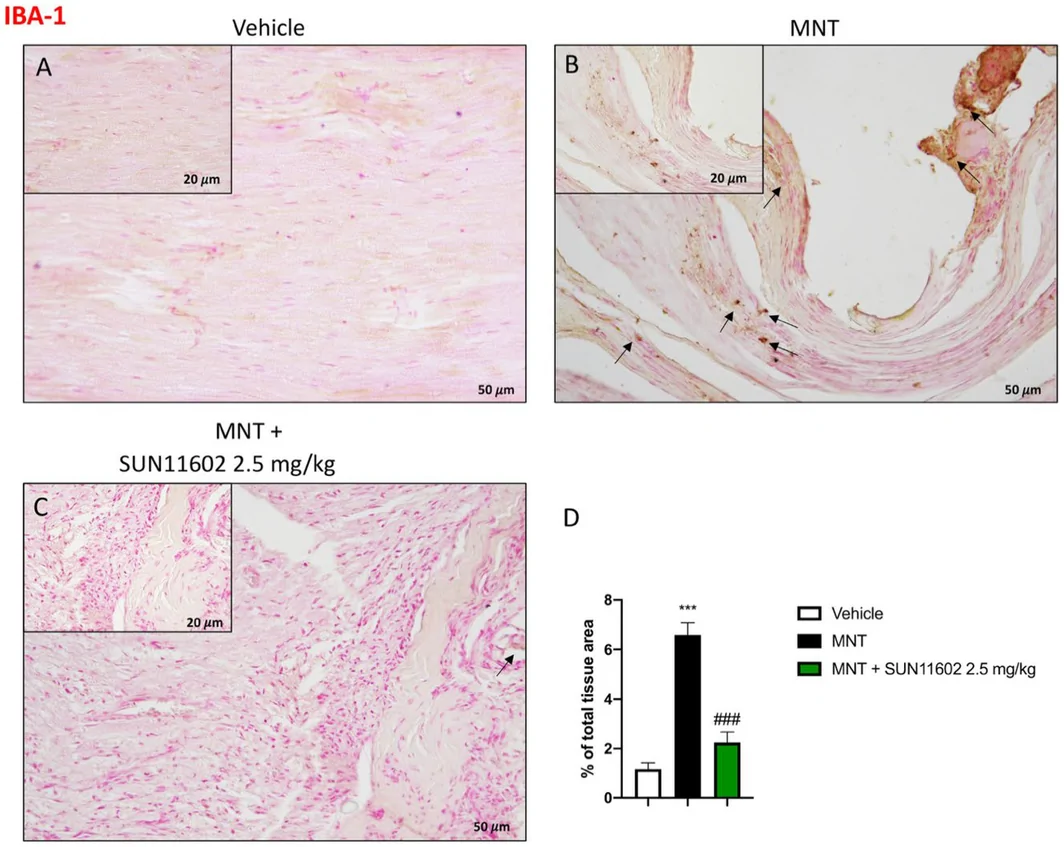
Immunohistochemistrystaining to evaluate IBA‐1 expression on sagittal sections of median nerve: vehicle group (A), MNT group (B), SUN11602‐treated group (C), score (D). The data are representative of at least three independent experiments. The sections were observed and photographed at 20× and 40× magnification. ***p < 0.001 vs. vehicle; ###*p* < vs. MNT group.

It is also known that nerve injury can activate resident macrophages [43, 44, 45]. To evaluate these cells' responses in the rat median nerve, we performed immunohistochemistry and immunofluorescence with antibodies against IBA‐1 and CD68 specifically located in macrophages. Our results showed an intense IBA‐1 immunoreactivity in the MNT group compared with the vehicle group as shown in Figure 4A,B (see score D). This upregulation of IBA‐1 induced by MNT was attenuated by treatment with SUN11602 at the dose of 2.5 mg/kg (Figure 4B,C, see score D).

**FIGURE 5:**
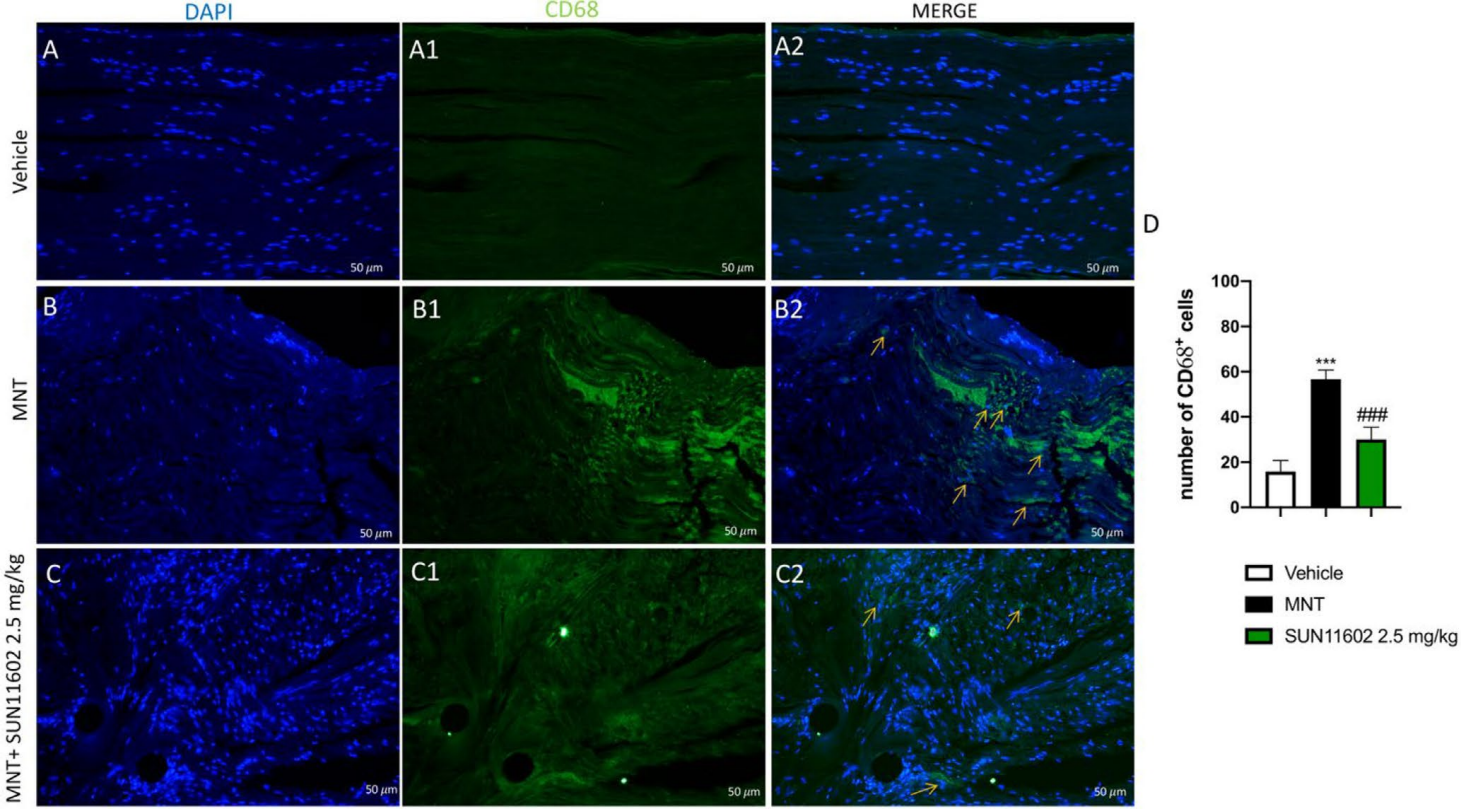
Immunofluorescence assay to evaluate CD68 expression on sagittal sections of median nerve: vehicle group (A), MNT group (B), SUN11602‐treated group (C), score (D). The data are representative of at least three independent experiments. The sections were observed and photographed at 20× magnification. ***p < 0.001 vs. vehicle; ###*p* < 0.001 vs. MNT group.

As stated, CD68 is a protein highly expressed by M1 macrophages that are recruited to the site of nerve injury, where they perform critical functions in the degradation of damaged nerve tissue, releasing cytokines and growth factors that modulate the inflammatory response and can influence the behaviour of other cells involved in nerve regeneration [46]. Our findings showed an increase in CD68 expression in the MNT group compared to the vehicle group (Figure 5A,B, see score D). These conditions were recovered with SUN11602 treatment at a dose of 2.5 mg/kg, as demonstrated by the reduction in CD68^+^ macrophage‐mediated inflammatory response (Figure 5C, see score D).

### 3.4 SUN11602 Treatment Promoted Schwann Cell Proliferation in Median Nerve Transection

**FIGURE 6:**
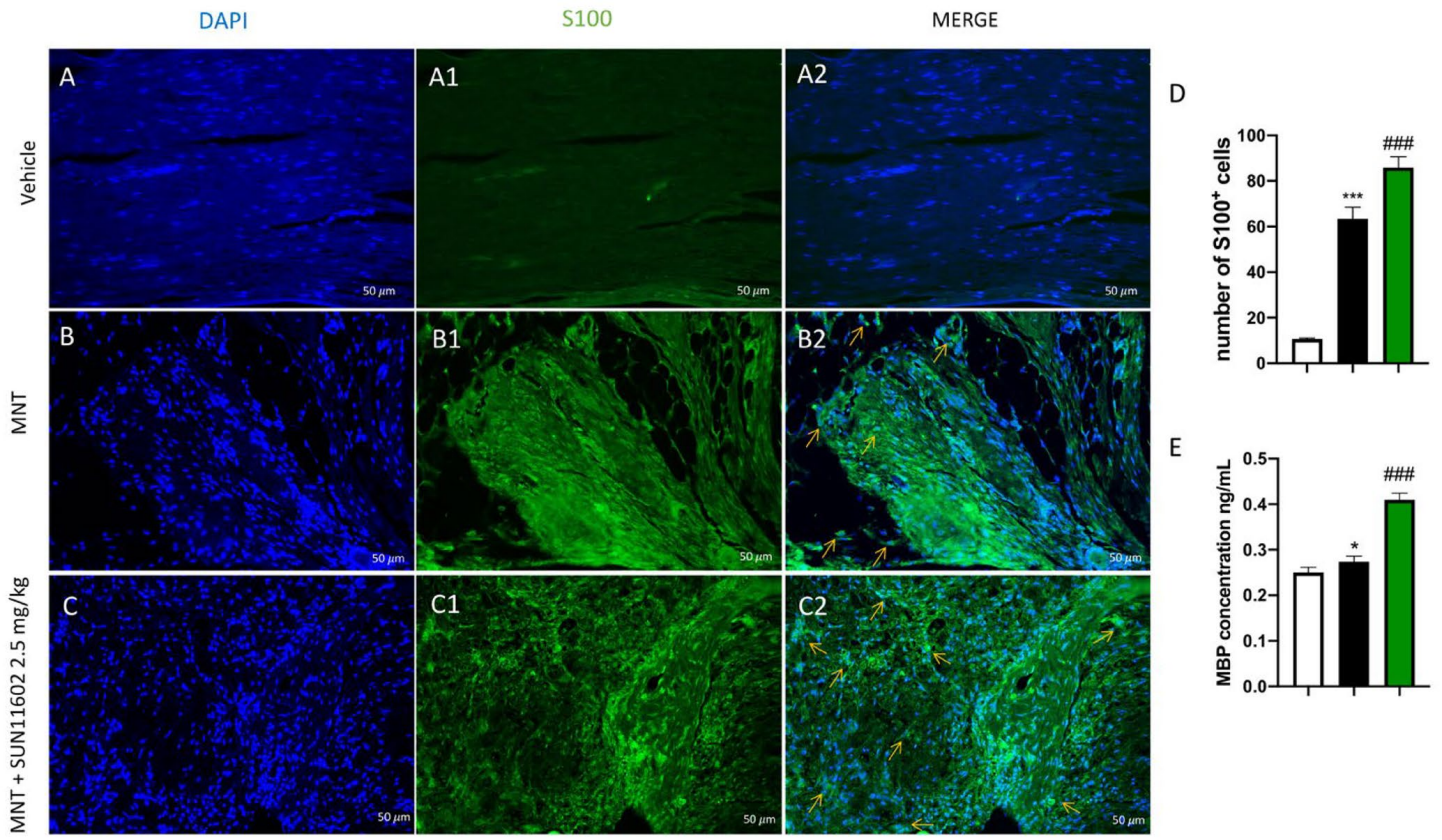
Immunofluorescenceassay to evaluate S100 expression on sagittal sections of median nerve: vehicle group (A), MNT group (B) SUN11602‐treated group (C), score (D). ELISA kit to evaluate MBP was performed (E). The data are representative of at least three independent experiments. The sections were observed and photographed at 20× magnification. *p < 0.05 vs. vehicle; ***p < 0.001 vs. vehicle; ### *p* < 0.001 vs. MNT.

During nerve regeneration, SCs help re‐establish the myelin sheath around the regenerated nerve fibres, clearing debris, secreting growth factors, driving axonal growth and facilitating efficient signal transmission [47, 48]. To elucidate the influence of SUN11602 treatment on the myelin sheath of nerve fibres, we labelled SCs with S100. We found that the stained areas of SCs in the SUN11602 group were more consistent and formed bonds compared to the MNT group, indicating that it promotes SC proliferation and median nerve myelinization (Figure 6A–C, see score D). To further support this observation, we quantified myelin basic protein (MBP) levels using an ELISA kit, which revealed a significant increase in MBP concentration in the SUN11602‐treated group compared to MNT, confirming enhanced myelin production at the molecular level (Figure 6E).

### 3.5 SUN11602 Treatment Reduced Median Nerve Demyelination and Fibrosis

**FIGURE 7:**
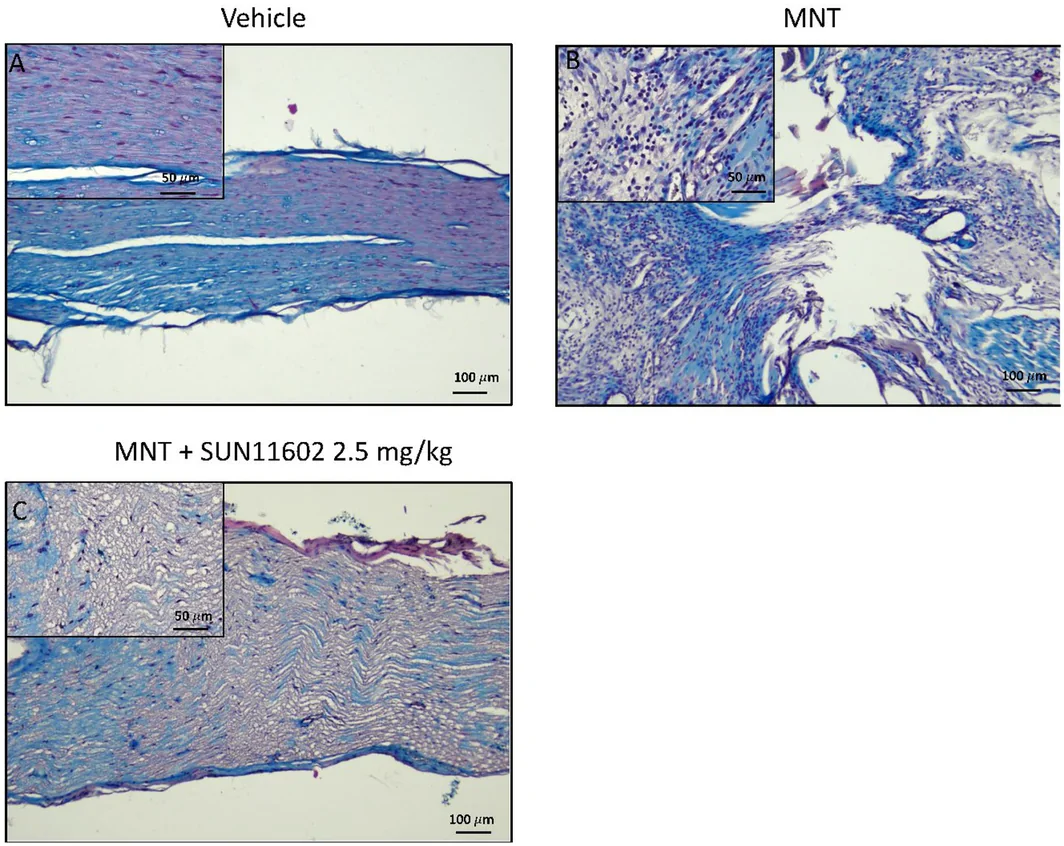
MCOLL staining on sagittal sections of median nerve: vehicle group (A), MNT group (B), SUN11602‐treated group (C). The data are representative of at least three independent experiments. The sections were observed and photographed at 10× and 20× magnification.

The analysis with MCOLL staining allowed us to observe the axonal regeneration process in the median nerve and simultaneously correlate all morphological parameters with the histochemical reaction for the myelin sheath (in blue) and collagen fibres (in red) [49]. Our results show signs of degeneration and a significant degree of demyelination followed by a marked fibrosis process with disorganized collagen fibres in the MNT group compared to the vehicle (Figure 7A,B). Treatment with SUN11602 at a dose of 2.5 mg/kg improved these processes leading to axonal remyelination and reorganization of collagen fibres (Figure 7C).

## 4 Discussion

Damage to the PNS can cause a variety of mechanical, ischaemic and inflammatory processes [50, 51, 52]. PNI can result from various causes leading to serious and long‐term functional disabilities [1, 53]. Restoration of the injured nerves requires a complex cellular and molecular response to reconnect the axons with their original targets [54]. Peripheral nerve injuries represent a significant clinical challenge due to the complexity of the regenerative process and the need for effective therapeutic interventions [53]. Nevertheless, there is no therapy for promoting nerve regeneration and functional recovery [55]. Supplementation with exogenous growth factors (GFs) is an emerging and versatile therapeutic strategy for promoting nerve regeneration and functional recovery [28]. In the present study, we used a rat PNI model to reveal the cellular and molecular mechanisms of the synthetic compound agonist of FGFR1 SUN11602, in modulating early nerve regeneration. Previous studies have shown that SUN11602 has neuroprotective effects against cognitive deficits and neuronal damage in an experimental model of Alzheimer's disease, depression and Parkinson's disease [56]. This compound has also been tested in a rat model of traumatic spinal cord injury showing neuroprotective activities similar to FGF [36]. Despite equivalent neuroprotective properties, there is a striking difference between SUN11602 and FGF. SUN11602 activates FGFR1, either directly or indirectly, to induce expression of calbindin required for neuroprotection, but it does not induce the proliferation of cells expressing endogenous FGFR1 [26]. Following peripheral nerve transection, Wallerian degeneration occurs and is characterized by structural changes distal to injury, in particular fragmentation of the myelin sheath, rearrangement of SCs into Bügner bands and macrophages infiltration [57]. Our study demonstrated that 7 days of treatment with SUN11602 exerted an anti‐inflammatory effect by reducing mast cells and macrophages infiltration. Mast cells are found close to peripheral nerves where they exercise a key role in orchestrating the inflammatory process from initiation through chronic activation. These cells represent important sources of inflammatory mediators and may have paramount roles in the development of neuropathic pain [41, 42]. Treatment with SUN11602 suppressed mast cells accumulation in MNT. Inflammation in PNI is primarily mediated by macrophages. Two types of macrophages are found in the injury site of PNS: resident macrophages and infiltrating macrophages [45, 58]. Resident macrophages are present in peripheral nerves and in ganglia under homeostatic conditions and can proliferate following injury or infection [59]. It has been seen that after a traumatic PNI resident macrophages contribute to nerve injury‐induced hypersensitivity [60, 61]. Treatment with SUN11602 reduced resident macrophages as shown by immunohistochemical expression of IBA1. Therefore, it could be assumed that pharmacological inhibition of resident macrophages by using a FGFR1 agonist was capable of improving neuropathic pain. Infiltrating macrophages are derived from bone marrow under injury stimulation [45]. At 3 h after injury, non‐resident macrophages infiltrate the damaged nerve and peak on Day 3, mainly with M1 phenotype. At 3–14 days, the infiltrating macrophages gradually convert from the M1 pro‐inflammation phenotype to the M2 anti‐inflammatory phenotype. Dynamic conversion of macrophage M1 to M2 sub‐phenotypes plays a pivotal role in regulating nerve regeneration after PNI [62, 63]. SUN11602 treatment decreased the expression of M1 macrophage marker CD68 demonstrating a potent anti‐inflammatory effect that could promote post‐injury nerve regeneration. Following nerve transection, SCs near the injury site of the proximal and distal nerve stumps undergo a rapid process of dedifferentiation to progenitor‐like cells that form tube‐like structures known as bands of Bügner which can act as ‘tunnels’ to guide the regrowing axons from the proximal nerve stump into the distal nerve stump [64, 65, 66]. On the other hand, demyelination and formation of scars represent unfavourable factors affecting the repair of PNI [67]. In our study on the experimental MNT model, SUN11602 oral administration for 7 days also promoted SC proliferation, reduced median nerve demyelination and fibrosis. All these results indicate that this compound promotes median nerve regeneration. In conclusion, the novel compound SUN11602 could like bFGF play a beneficial role in neuroprotection, anti‐inflammation and neurite outgrowth in an experimental model of early peripheral regeneration. While our findings support a neuroprotective role of SUN11602 in promoting SC proliferation and preserving myelin integrity following PNI, several limitations should be acknowledged. First, the observed attenuation of myelin degradation should not be interpreted as a global inhibition of Wallerian degeneration, which is a physiologically required process for successful regeneration. Rather, we hypothesize that SUN11602 may selectively reduce secondary or excessive demyelination in spared fibres, a distinction that requires further validation through ultrastructural analysis and time‐course studies of myelin clearance. Furthermore, all experimental evaluations in this study were conducted at a single early time point, 7 days post‐suture, corresponding to the initial phase of Wallerian degeneration and SC activation. At this stage, axonal regeneration and remyelination are only beginning and remain incomplete. As such, our findings primarily reflect early molecular and cellular responses to SUN11602 treatment rather than long‐term regenerative outcomes. Future studies should include extended time points to monitor axonal regrowth, remyelination dynamics and functional recovery over time in order to more comprehensively assess the therapeutic potential of SUN11602 in nerve repair. In particular, the incorporation of functional and electrophysiological evaluations will be essential to correlate the observed histological improvements with meaningful recovery of nerve function.

## 5 Conclusions

In the present study, we demonstrated that oral administration of SUN11602 in a rat model of MNT promotes early regenerative processes. Specifically, SUN11602 treatment preserved nerve tissue architecture, reduced mast cell accumulation and macrophage‐mediated inflammation, enhanced SC activity and limited demyelination and fibrosis. Taken together, these findings support a neuroprotective and pro‐regenerative role of SUN11602 during the early phase following PNI. However, these results are limited to a single early time point and are primarily based on histological and molecular analyses. Therefore, further studies are required to determine whether these early effects can translate into sustained axonal regeneration and functional recovery over time.

Future investigations should include extended follow‐up periods to monitor axonal regrowth, remyelination dynamics and long‐term outcomes. In particular, the integration of functional and electrophysiological assessments will be essential to correlate the observed histological improvements with meaningful restoration of nerve function. Moreover, evaluating SUN11602 in combination with alternative nerve repair strategies, such as nerve conduits, biomaterials or surgical approaches beyond direct suturing, will be crucial to better mimic complex clinical conditions and enhance the translational relevance of this approach.

In conclusion, SUN11602 represents a promising pharmacological candidate for modulating early events in peripheral nerve regeneration, warranting further investigation in long‐term and functionally oriented studies.

## Author Contributions

**Giovanna Casili:** writing – original draft. **Igor Papalia:** conceptualization. **Alessio Ardizzone:** writing – review and editing, methodology. **Rossella Basilotta:** writing – original draft, methodology. **Alfio Luca Costa:** supervision. **Paolo Titolo:** supervision. **Emanuela Esposito:** supervision, conceptualization. **Michele Rosario Colonna:** conceptualization, supervision. **Mariarosaria Galeano:** conceptualization, writing – review and editing.

## Funding

The authors have nothing to report.

## Conflicts of Interest

The authors declare no conflicts of interest.

## Data Availability

The data that support the findings of this study are available from the corresponding author upon reasonable request.
